# Comparison of Del Nido and histidine-tryptophan-ketoglutarate cardioplegia solutions: an animal study with prolonged ischaemia

**DOI:** 10.3389/fcvm.2024.1457770

**Published:** 2024-12-17

**Authors:** Alexandro Hoyer, Maja-Theresa Dieterlen, Jagdip Kang, Hanna Oetzel, Karoline Wiesner, Kristin Klaeske, Philipp Kiefer, Susann Oßmann, André Ginther, Martin Kostelka, Suzanne de Waha, Michael A. Borger

**Affiliations:** Heart Centre Leipzig, University Clinic of Cardiac Surgery, HELIOS Clinic, University Leipzig, Leipzig, Germany

**Keywords:** Del Nido solution, Bretschneider solution, histidine-tryptophan-ketoglutarate solution, custodiol, single-shot cardioplegia, animal model, ischaemia

## Abstract

**Objective:**

Myocardial protection is important for a successful procedure cardiac surgery, and the key element of myocardial protection is cardioplegia. We compared Del Nido cardioplegia (DN) and Bretschneider histidine-tryptophan-ketoglutarate cardioplegia (HTK) regarding cardioprotective effects in a porcine model of prolonged ischaemia.

**Methods:**

Landrace pigs weighing 50–60 kg were randomized to receive either DN (*n* = 9) or HTK (*n* = 9). All pigs underwent cardiac arrest for 90 min followed by 120 min of reperfusion/convalescence. A detailed set of laboratory, histological and functional parameters was acquired at baseline, during cardiac arrest and following reperfusion/convalescence.

**Results:**

Pressure-volume measurements revealed better systolic and diastolic left ventricular performance in DN as compared to HTK (both *p* < 0.05). Haemoglobin decreased after application of the cardioplegic solution. The decrease was more pronounced in the HTK group than in the DN group (*p* < 0.01). In contrast to DN, sodium (*p* < 0.01) and chloride levels (*p* < 0.05) were significantly decreased in the HTK group after initiation of CPB and remained decreased after reperfusion. The number of animals requiring defibrillations to restore sinus rhythm significantly differed between the groups [HTK: 100% (*n* = 9/9) vs. DN: 44.4% (*n* = 4/9), *p* = 0.03]. Expression of ICAM-1 as a marker of endothelial dysfunction was lower in the DN group compared to the HTK group (*p* = 0.02). Histological evaluation, oxidative and nitrosative stress, mitochondrial membrane integrity and apoptosis markers were comparable between DN and HTK groups (all *p* > 0.05).

**Conclusions:**

In this porcine model with prolonged ischaemia, DN was superior to HTK in terms of haemoglobin levels, blood electrolytes, spontaneous return of sinus rhythm, left ventricular function, and endothelial injury. Histomorphological parameters indicative of ischaemia/reperfusion injury, oxidative stress and mitochondrial function as well as apoptosis-inducing factors did not differ.

## 1 Introduction

Myocardial protection is of utmost importance for a successful procedure cardiac surgery, and the key element of myocardial protection is cardioplegia (1, 2). Left ventricular (LV) function post-cardioplegic arrest is a clinically important variable, since low cardiac output syndrome post-cardiac surgery is associated with worse perioperative and long-term outcomes (3). In the last decades, the use of the single-shot Bretschneider histidine-tryptophan-ketoglutarate cardioplegia (HTK) has been widely adopted in adult cardiac surgery (4). An alternative to HTK is Del Nido cardioplegia (DN), which was primarily designed for paediatric cardiac surgery. A randomized clinical trial enrolling patients aged ≤12 years demonstrated a beneficial safety profile of DN in comparison to HTK with improved preservation of cardiac index, less release of troponin I, and shorter hospital stays (5).

In recent years, DN has been increasingly used in adult patients, despite its original designed purpose for paediatric patients. Furthermore, inconsistent results regarding cardioprotective effects of DN and HTK have been observed, mostly in retrospective studies (6–11). In the absence of adequately randomized clinical trials and evidence mainly limited to retrospective studies prone to inherent selection bias, the effects with respect to the serological, functional, and histological impact of DN compared to HTK have not been fully elucidated.

The aim of the current study was therefore to compare the effects of DN vs. HTK on post-myocardial ischaemia LV function, haemoglobin levels, blood electrolytes, histomorphological parameters indicative of ischaemia/reperfusion injury, oxidative stress, mitochondrial and endothelial function as well as apoptosis-inducing factors using a porcine model of prolonged ischaemia.

## 2 Materials and methods

### 2.1 Animals and surgical intervention

**Figure 1:**
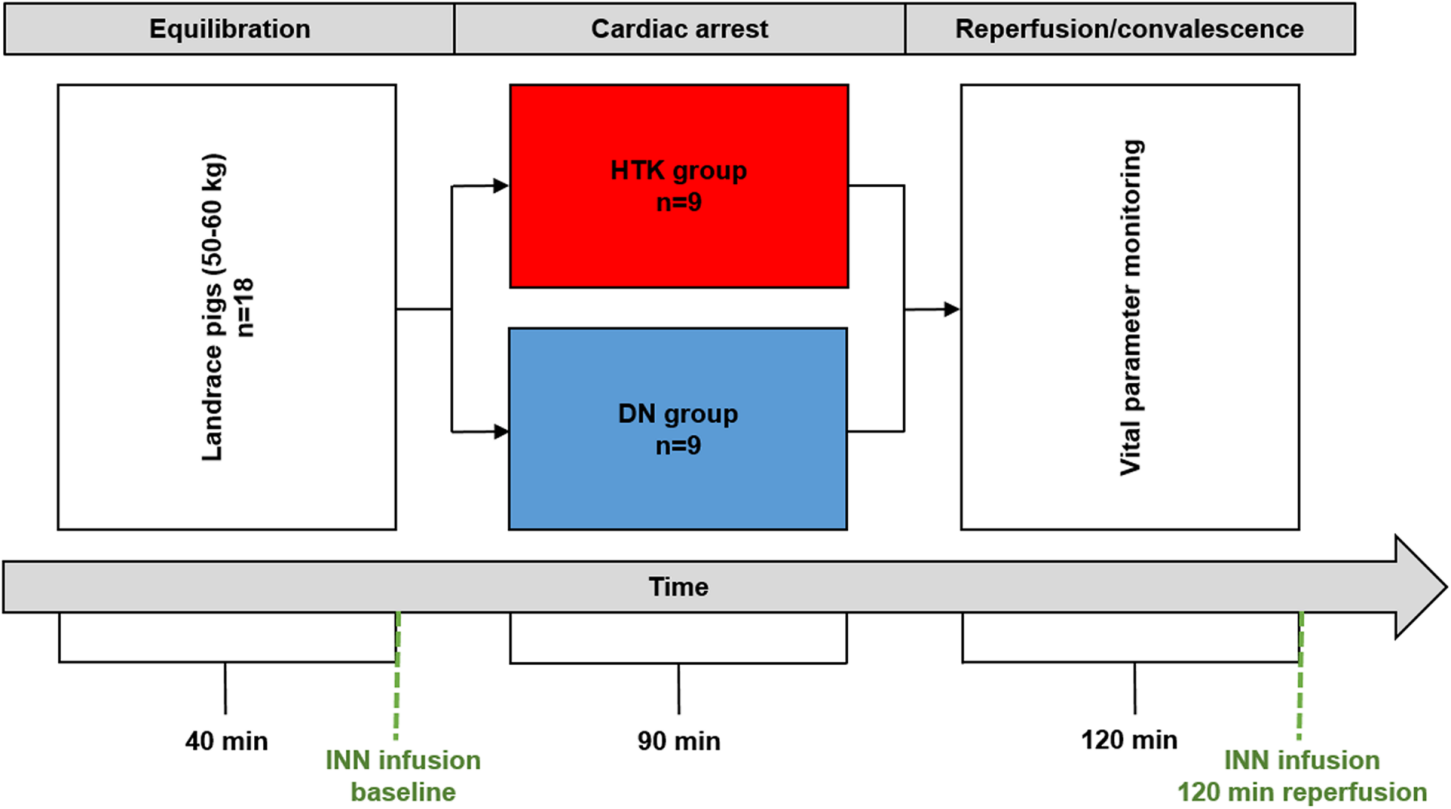
Study design. DN, Del Nido cardioplegia; HTK, Bretschneider histidine-tryptophan-ketogluterate cardioplegia; INN, norepinephrine.

The study was approved by the local animal welfare agency of the University of Leipzig (TVV 23/19) and conducted in accordance with guideline 2010/63/EU. The study design is shown in Figure 1. The original base solution for DN is Plasmalyte A, which does not have a market authorization in Germany. Therefore, a modified DN was used (Jonosteril Free Flex 1,000 ml, potassium chloride 14.9%, sodium bicarbonate 8.4%, magnesium sulphate 50%, lidocain 2%, mannitol 15% cardioplegia:blood ratio 4:1). Animals were randomly assigned (*n* = 9 per group) to receive either HTK (Dr. Franz Köhler Chemie, Bensheim, Germany) or DN. Details on anaesthesia, surgical technique, extracorporeal circulation and perfusion are summarized in the Supplementary Material. In brief, animals underwent anaesthesia and mechanical ventilation followed by sternotomy and establishment of CPB. Before cross-clamping of the aorta and infusion of the cardioplegic solution (application volume: HTK 1,800 ml, DN 1,300 ml) all animals were equilibrated for 40 min. This period comprised induction of anaesthesia, preparing surgical access and hemodynamical stabilization. A standardized duration of the equilibration period was defined to improve comparability of the study groups. The estimated duration of 40 min corresponds to the duration for anaesthesia induction, surgical access and hemodynamical stabilization in human and could be realized with the expertise of experienced veterinarians. Baseline measurements were performed after hemodynamical stabilization at the end of the 40 min equilibration period. Following the application of the cardioplegic solution, the hearts remained arrested for 90 min at 34°C body temperature. The aortic cross-clamp was removed after 90 min of ischaemia. After 30 min of reperfusion, the animals were weaned from CPB within a time frame of 15 min. The total time after the removal of the cross-clamp was determined as reperfusion period. The reperfusion period was set to 120 min. Apart from the cardioplegia solutions used, there were no differences in terms of anaesthesia and surgery.

### 2.2 Randomization and blinding

Simple randomization was performed securing the generation of an unpredictable allocation of the animals to each group. Blinding of the surgical team was not possible due to the differences in the preparation of the cardioplegic solutions. All other investigators involved in data analyses were blinded to treatment allocation.

### 2.3 Pressure-volume measurements

Left ventricular (LV) performance was measured by obtaining pressure-volume loops using a 7F conductance catheter (CD Leycom, Zoetermeer, the Netherlands) via the common carotid artery before initiation of CPB (baseline) and 120 min after the removal of the cross-clamp. The catheter was removed before initiation of CPB and reinserted through the existing vascular access after removal of the cross clamp. The catheter was placed using fluoroscopy to ensure proper placement and reproducibility after removal. Before reinsertion, the catheter was recalibrated and after placement hypertonic saline injection was performed to account for myocardial edema. To assess the accuracy of the measurements of stroke volume and cardiac output, thermodilution measurements using a PiCCO® catheter (PULSION Medical Systems, Feldkirchen Germany) placed in the femoral artery were obtained.

All measurements at baseline and 120 min after the removal of the cross-clamp were performed under a continuous rate infusion of norepinephrine (INN) at either 4 µg/kg or 8 µg/kg body weight to standardize the reaction of the cardiovascular system to INNs. INN infusion following cardiac arrest and CPB support is necessary in the majority of interventions in both human and pig due to its positive inotropic, chronotropic and dromotropic effects. To compare cardiac function and LV performance after necessary INN infusion due to CPB and cardiac arrest, it is important to have a valide reference prior to cardiac arrest. Thus, our study design included LV performance measurements with defined INN doses at baseline.

As systolic properties, preload recruitable stroke work (PRSW) and the index of end-systolic pressure-volume relationship extrapolated volume at 100 mmHg (V_Pes100_) were assessed. As diastolic properties, Tau (rate of pressure decay during isovolumetric relaxation) and index of the end-diastolic pressure-volume relationship extrapolated volume at 10 mmHg (V_Ped10_) were measured.

### 2.4 Sample preparation

Myocardial biopsies of the LV (*n* = 10) and serum samples were obtained at baseline, after 90 min of ischaemia, and after 120 min following reperfusion. After 90 min of ischaemia, myocardial transmural biopsies from the LV were harvested using a 18G biopsy gun (Möller Medical GmbH, Fulda, Germany). Right ventricular (RV) biopsies (*n* = 5) were taken after 120 min following reperfusion. Finally, biopsies of the left anterior descending artery (LAD) were procured. Biopsies were (i) snap-frozen in liquid nitrogen and stored at −80°C for biochemical analyses, or (ii) transferred to 0.9% NaCl for further analysis of the mitochondrial membrane potential, or (iii) fixed with 4% formaldehyde in phosphate-buffered saline (pH 7.4) for histological analysis. Blood gas analyses including measurements of pH, lactate and electrolyte concentrations were performed in blood samples withdrawn at baseline, after aortic cross clamping, after 90 min of ischaemia and after 120 min reperfusion using an ABL 90 flex (Radiometer, Willich, Germany). Serum samples were obtained at baseline and after 120 min reperfusion. Serum samples were centrifuged at 2,000*g for 20 min at room temperature before aliquoting and storing at −20°C.

### 2.5 Histological evaluation

**Figure A1:**
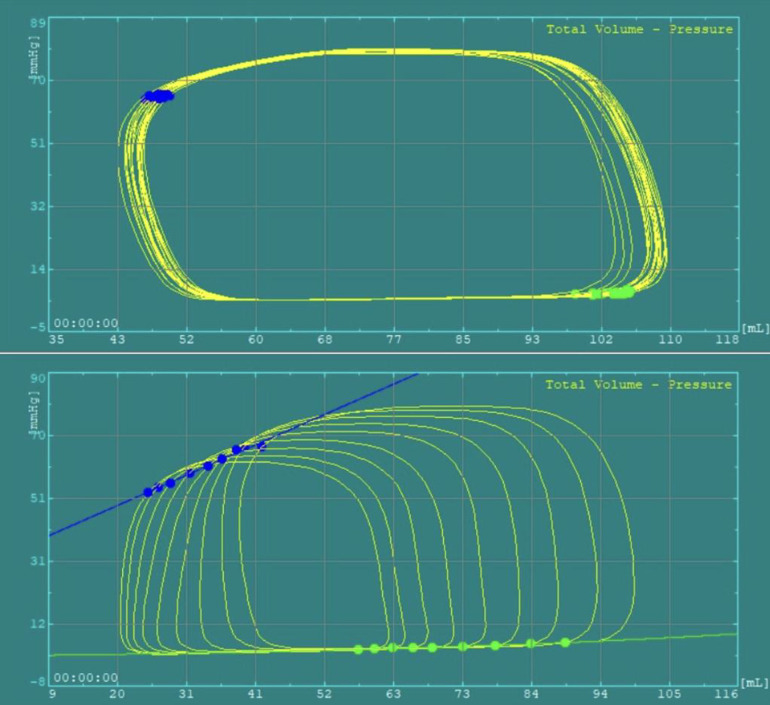
Recorded pressure volume loops during breathhold (upper) and under breathold and occlusion of the inferior vena cava.

Following formaldehyde-fixation, LV and RV as well as LAD biopsies were embedded in paraffin and cut into 3 µm histological sections. Haematoxylin/eosin staining of myocardial sections was performed as described previously (12) to evaluate the colourability of cell borders and nuclei as well as the presence of oedema and cross striation of cardiomyocytes (Appendix Figure A1).

Immunohistochemical stainings of myocardial cross-sections of LV and RV for hypoxia-inducible factor 1α (HIF-1α), nitrotyrosin and the apoptosis-inducing factor (AIF) were performed as described by Feirer et al. to evaluate oxidative and nitrosative stress as well as caspase-independent apoptosis (13). Similarly, immunohistochemical staining of LAD sections was performed using a rabbit anti-endothelial nitric oxid synthetase (eNOS) antibody (1:100 dilution; Invitrogen, Waltham, MA, USA) and a mouse anti-intracellular adhesion molecule-1 (ICAM-1) (1:250 dilution; antibodies-online GmbH, Aachen, Germany). The histological evaluation at 200 × magnification was performed using the Axio Plan 2 microscope (Carl Zeiss AG, Jena, Germany) AxioVision Release 4.8.2 SP3 (Carl Zeiss AG, Jena, Germany) and image 2.0.0 software (US National Institutes of Health, Bethesda, MD, USA).

Mitochondrial membrane integrity was assessed using the JC-1 Mitochondrial Membrane Potential Assay (Cayman Chemical, Ann Arbor, Michigan, USA) (14). The BD LSR II cytometer and the FACS Diva 6.1.3 software (both from BD, Franklin Lakes, NJ, USA) were used for a flow cytometric quantification of the green and red fluorescence of the 5,5,6,6'-tetrachloro-1,1',3,3' tetraethylbenzimi-dazoylcarbocyanine iodide (JC-1) dye. 10,000 events were analyzed per sample.

### 2.6 Serological analyses

Troponin I and creatine kinase MB (CK-MB) were quantified in serum samples obtained at 120 min reperfusion using enzyme-linked immunosorbent assays (ELISA) for troponin I and CK-MB (both antibodies-online GmbH, Aachen, Germany), Infinite 200 PRO microplate reader (Tecan Trading AG, Männedorf, Switzerland), and i-control™ 1.12 software (Tecan Trading AG, Männedorf, Switzerland). Serum concentrations of angiotensin I and angiotensin II were assessed at baseline and after 120 min reperfusion using Porcine Angiotensin I and Porcine Angiotensin II ELISA (Mybiosource, San Diego, CA, USA), Infinite 200 PRO microplate reader (Tecan Trading AG, Männedorf, Switzerland), and i-control™ 1.12 software (Tecan Trading AG, Männedorf, Switzerland).

### 2.7 Statistical analysis

Categorical variables are expressed as frequencies and percentages. Continuous variables are expressed as mean ± standard deviation. Continuous variables were compared using Student's *t*-test in case of normal distribution or with Mann-Whitney U test in case of non-normal distribution. Group comparisons of categorical variables were performed using the *χ*^2^ test for frequencies greater than 5 or using Fisher's exact test for frequencies lower or equal 5. *P* values ≤ 0.05 were considered significant. Statistical analyses were performed with SPSS Statistics 28 software (IBM, Armonk, NY, USA).

## 3 Results

### 3.1 LV performance and spontaneous return to sinus rhythm

Independent of the adminestred cardioplegia, all animals required continuous norepinephrine support after termination of CPB to maintain adequate mean arterial pressures >50 mmHg. Baseline values and representative pressure volume loop were displayed in Appendix Table A1 and Figure A1. Under INN administration, there were no significant differences with respect to heart rate, systolic and diastolic blood pressure, systolic and diastolic pulmonary artery pressure, central venous pressure, cardiac index, and systemic vascular resistance during baseline and after 120 min reperfusion between the two groups (all *p* > 0.05). During reperfusion, the number of animals requiring defibrillations to restore sinus rhythm significantly differed between the groups [HTK 100% (*n* = 9/9) vs. DN 44.4% (*n* = 4/9), *p* = 0.03]. Results of pressure-volume loops are displayed in Table 1. Results of systolic performance assessed by ΔV_Pes100_ was in favour of DN. Further, diastolic measurements were improved by DN compared to HTK (ΔV_Ped10_ at 4 µg/kg INN, ΔTau with 8 µg/kg INN).

**Table 1:** Pressure-volume measurements.

	HTK (*n* = 9)	DN (*n* = 9)	*p* value	HTK (*n* = 9)	DN (*n* = 9)	*p* value
	4 µg/kg INN			8 µg/kg INN		
Systolic measurements						
ΔV_Pes100_	−97 ± 105 ml	−23 ± 27 ml	0.03	−118 ± 99 ml	−46 ± 51 ml	0.04
ΔPRSW	45 ± 45 mmHg	25 ± 24 mmHg	0.16	50 ± 47 mmHg	27 ± 29 mmHg	0.12
Diastolic measurements						
ΔV_Ped10_	−298 ± 369 ml	100 ± 206 ml	0.03	−246 ± 362 ml	−40 ± 232 ml	0.10
ΔTau	−2.0 ± 2.3 ms	0.3 ± 3.3 ms	0.09	−3.1 ± 3.5 ms	0.5 ± 2.1 ms	0.01

Data are presented as Δ of baseline measurements before the initiation of cardiopulmonary bypass and measurements 120 min cross-clamp removal under INN doses of 4 µg/kg and 8 µg/kg. Values are mean ± standard deviation. DN, Del Nido cardioplegia; HTK, Bretschneider histidine-tryptophan-ketogluterate cardioplegia; INN, norepinephrine; PRSW, preload recruitable stroke work; V_Pes100,_ index of end-systolic pressure-volume relationship extrapolated volume at 100 mmHg; V_Ped10_, index of the end-diastolic pressure-volume relationship extrapolated volume at 10 mmHg; Tau, rate of pressure decay during isovolumetric relaxation.

### 3.2 Haemoglobin, electrolytes and markers of myocardial injury

Haemoglobin levels were similar at baseline (HTK 5.8 ± 0.4 mmol/L vs. DN 5.6 ± 0.3 mmol/L; *p* = 0.43) and decreased after administration of the cardioplegic solution (HTK 3.7 ± 0.4 mmol/L vs. DN 4.2 ± 0.3 mmol/L). The decrease was more pronounced in the HTK group than in the DN group (*p* < 0.01).

**Figure 2:**
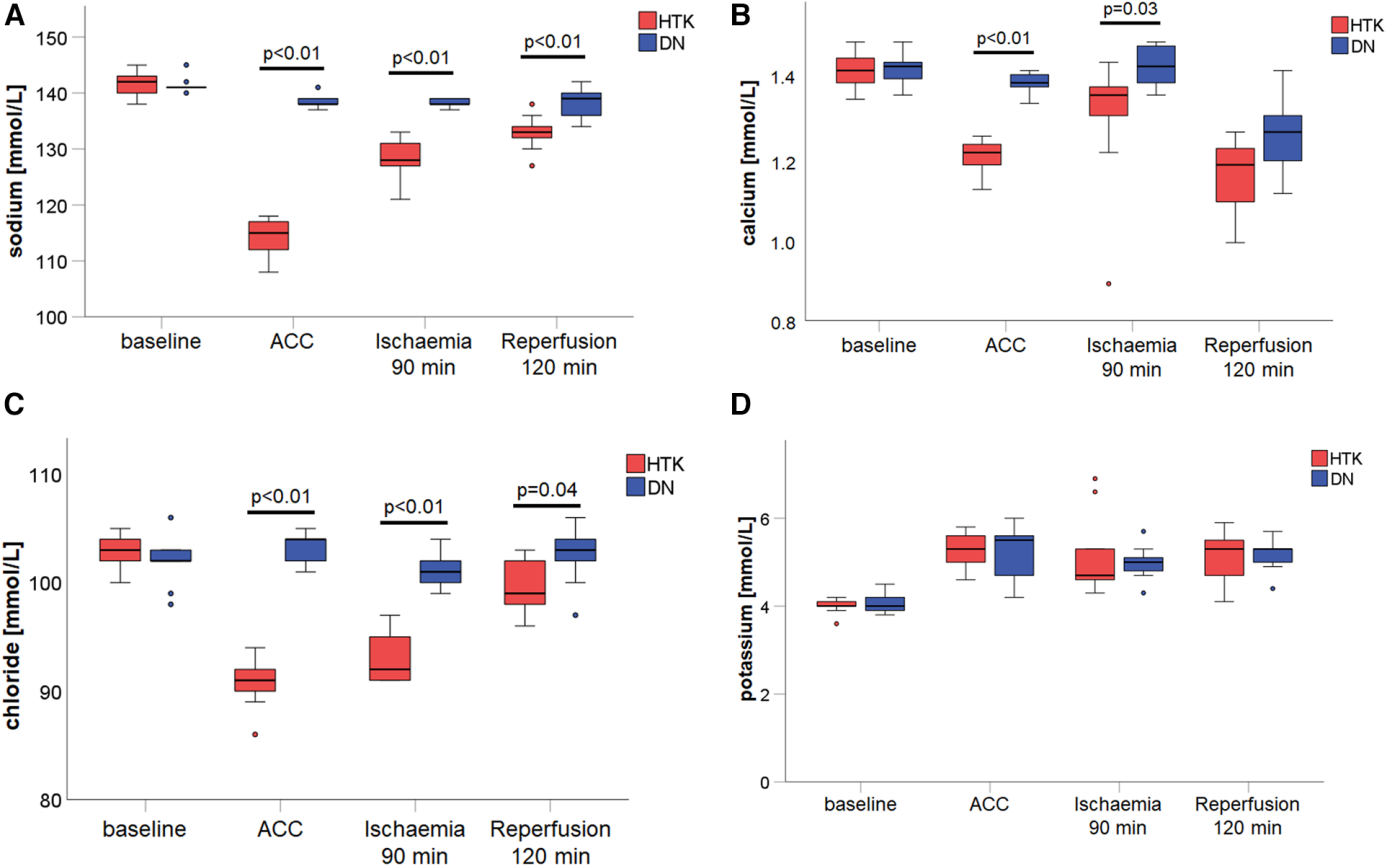
Blood concentrations of sodium **(A)**, calcium **(B)**, and chloride **(C)** and potassium **(D)** at baseline, before and after 90 min cardiac arrest and after 120 min reperfusion. The whiskers of the plot extend from the 10th percentile to the 90th percentile. The line inside the box is the median and the top and bottom of the box represent the 1st and 3rd quartiles. Dots indicate outliers. ACC, aortic cross-clamp; DN, Del Nido cardioplegia; HTK, Bretschneider histidine-tryptophan-ketogluterate cardioplegia.

Changes in electrolytes levels during ischaemia and after 120 min reperfusion were present in the HTK group compared to the DN group (Figures 2A–D). Sodium (*p* < 0.01) and chloride levels (*p* < 0.05) were decreased in the HTK group after initiation of CPB, and remained decreased after reperfusion. In contrast to DN, calcium (*p* < 0.05) decreased in the HTK group during ischaemia, but returned to normal levels during reperfusion. Potassium remained stable and did not differ between groups.

At baseline, troponin I (HTK 428 ± 477 pg/ml vs. DN 225 ± 166 pg/ml, *p* = 0.26) and CK-MB (HTK 166 ± 212 pg/ml vs. DN 134 ± 208 pg/ml, *p* = 0.75) were comparable. After 120 min reperfusion, there were no significant differences with respect to troponin I (HTK 467 ± 112 pg/ml vs. DN 360 ± 87 pg/ml, *p* = 0.46) and CK-MB (HTK 172 ± 38 mU/ml vs. DN 114 ± 33 mU/ml, *p* = 0.27) (Table 2).

**Table 2:** Myocardial, mitochondrial, and endothelial injury.

	HTK (*n* = 9)	DN (*n* = 9)	*p* value
Myocardial injury			
Troponin I	467 ± 112 pg/ml	360 ± 87 pg/ml	0.46
CK-MB	172 ± 38 mU/ml	114 ± 33 mU/ml	0.27
Mitochondrial injury			
JC-1 ratio after 90 min ischaemia	38 ± 11%	26 ± 4%	0.37
JC-1 ratio after 120 min ischaemia	26 ± 3%	32 ± 5%	0.32
Endothelial injury			
ICAM-1	11.8 ± 9.4%	3.3 ± 5.7%	0.02
eNOS	22.4 ± 32.1%	10.2 ± 13.2%	0.17

Data are represented as mean ± standard deviation. CK-MB, creatine kinase MB; DN, Del Nido cardioplegia; eNOS, endothelial nitric oxid synthetase; HTK, Bretschneider histidine-tryptophan-ketogluterate cardioplegia; ICAM-1, intracellular adhesion molecule-1.

### 3.3 Histological evaluation of myocardial tissue

The colourability of cell borders and nuclei of cardiomyocytes was determined in the LV and RV as a histological sign of myocardial damage. Results are displayed in Table 3. Colourability of cell borders (LV: *p* = 0.34; RV: *p* = 1) and nuclei (LV: *p* = 0.84; RV: *p* = 1) did not differ between the HTK and the DN group. The presence of cross striation of cardiomyocytes, which are visible signs for intact myofilaments in cardiomyocytes, were comparable between the HTK and the DN group in the LV (*p* = 0.39) and the RV (*p* = 0.54). After 120 min reperfusion, LV oedema was observed in 89% of the biopsies in the HTK group and in 82% of the biopsies in the DN group (*p* = 1). RV oedema was documented in all (100%) biopsies in the HTK group and in 91% of the biopsies in the DN group (*p* = 1). Cell infiltration, mainly by immune cell populations, were comparable between the HTK and the DN group (LV: HTK 78% vs. DN: 64%, *p* = 0.84; RV: HTK 89% vs. DN: 91%, *p* = 1).

**Table 3:** Evaluation of LV and RV biopsies.

	LV biopsies		RV biopsies			
	HTK (*n* = 9)	DN (*n* = 9)	*p* value	HTK (*n* = 9)	DN (*n* = 9)	*p* value
Histological evaluation						
Colourability of cell borders			0.45			1.00
0–30%	4 (44%)	5 (56%)		4 (44%)	5 (56%)	
30–70%	5 (56%)	3 (33%)		5 (56%)	4 (44%)	
>70%	0 (0%)	1 (11%)		0 (0%)	0 (0%)	
Colourability of nuclei			0.62			1.00
0–30%	0 (0%)	0 (0%)		0 (0%)	0 (0%)	
30–70%	7 (78%)	5 (56%)		8 (89%)	8 (89%)	
>70%	2 (22%)	4 (44%)		1 (11%)	1 (11%)	
Cross striation of cardiomyocytes			0.26			1.00
0–30%	8 (89%)	5 (56%)		7 (78%)	6 (67%)	
30–70%	1 (11%)	3 (33%)		2 (22%)	3 (33%)	
>70%	0 (0%)	1 (11%)		0 (0%)	0 (0%)	
Oedema	8 (89%)	8 (89%)	1.00	9 (100%)	8 (89%)	1.00
Cell infiltration	7 (78%)	6 (67%)	1.00	8 (89%)	8 (89%)	1.00
Oxidative stress						
HIF-1α translocation	29.0 ± 6.2%	24.6 ± 2.3%	0.51	33.1 ± 7.4%	23.7 ± 2.2%	0.25
Apoptosis						
AIF cytosolic release	16.3 ± 5.1%	21.2 ± 6.9%	0.59	18.5 ± 4.4%	20.2 ± 4.8%	0.79
Cyt C cytosolic release	2.4 ± 1.1%	2.4 ± 0.8%	0.44	2.7 ± 1.5%	2.5 ± 0.8%	0.38
Nitrosative stress						
Nitrotyrosine expression	0.35 ± 0.26%	0.10 ± 0.04%	0.35	0.20 ± 0.16%	0.27 ± 0.16%	0.75

Values are *n* (%) or mean ± standard deviation. AIF, apoptosis-inducing factor; Cyt C, cytochrome C, DN, Del Nido cardioplegia; HIF-1α, hypoxia-inducible factor 1α; HTK, Bretschneider histidine-tryptophan-ketogluterate cardioplegia; LV, left ventricular; RV, right ventricular.

### 3.4 Oxidative stress and mitochondrial membrane integrity

The translocation of the transcription factor HIF-1α into the nucleus is an indicator for changes of tissue oxygen supply and oxidative stress (Table 3). After 120 min reperfusion, HIF-1*α* translocation could be detected in 29.0 ± 6.2% of myocardial cells of the LV in the HTK group and in 24.6 ± 2.3% in the DN group (*p* = 0.51). Similarly, HIF-1α translocation in myocardial cells of the RV did not differ between the groups (HTK: 33.1 ± 7.4%, DN: 23.7 ± 2.2%, *p* = 0.25).

Staining with JC-1 dye allows discrimination between mitochondria with intact or impaired membrane potential. A ratio of green (= impaired membrane potential) and red fluorescence (= intact membrane potential) visualizes mitochondrial damage (Table 1). The degree of mitochondrial damage after 90 min ischaemia (*p* = 0.37) and after 120 min reperfusion (*p* = 0.32) was comparable between the HTK and the DN group.

### 3.5 Rate of apoptosis induction

The release of AIF and cytochrome C from mitochondria into the cytosol is an important step in apoptosis induction (Table 3). The cytosolic release of AIF did not differ between groups (LV: *p* = 0.59, RV: *p* = 0.79). Similar findings were documented regarding the release of cytochrome C into the cytosol of cardiomyocytes (LV: *p* = 0.44, RV: *p* = 0.38).

### 3.6 Nitrosative stress and endothelial injury

Nitrosative stress is an indicator for impaired oxygen metabolism associated with ischaemia/reperfusion injury and can be detected by the expression of 3-nitrotyrosine (Table 3). Nitrotyrosine expression was quantified in cardiac biopsies after 120 min reperfusion and did not differ groups (LV: *p* = 0.35, RV: *p* = 0.75).

Endothelial cells of the LAD were stained for eNOS and ICAM-1 expression to evaluate the grade of endothelial injury after 120 min reperfusion (Table 1). The percentage of endothelial cells with ICAM-1 expression was lower in the DN group compared to the HTK group (3.3 ± 5.7% vs. 11.8 ± 9.4%, *p* = 0.02), whereas the percentage of eNOS expressing cells did not differ (10.2 ± 13.2% vs. 22.4 ± 32.1%, *p* = 0.17).

Comparing levels at baseline and after 120 min reperfusion, serum angiotensin I decreased in both groups (HTK: baseline 2.65 ± 0.83 ng/ml, 120 min reperfusion 1.76 ± 0.36 ng/ml, *p* < 0.01; DN: baseline 2.52 ± 0.96 ng/ml, 120 min reperfusion 1.71 ± 0.27 ng/ml, *p* = 0.01), but did not differ between the groups (*p* = 0.82). Levels of angiotensin II at baseline and after 120 min reperfusion were comparable either in the HTK (baseline 151 ± 118 ng/L, 120 min reperfusion 179 ± 121 ng/L, *p* = 0.27) or in the DN group (baseline 158 ± 44 ng/L, 120 min reperfusion 158 ± 44 ng/L, *p* = 0.99) and did not differ between both groups (*p* = 0.41).

## 4 Discussion

The present study of a porcine model with prolonged ischaemia demonstrates superiority of DN in comparison to HTK with respect to blood electrolytes, haemoglobin levels, spontaneous return of sinus rhythm, LV function, and endothelial injury. Histological evaluation, oxidative and nitrosative stress, mitochondrial membrane integrity and apoptosis-inducing factors did not differ between groups.

Cardioplegia is a key component for a successful procedure. An ideal cardioplegia solution should offer optimized myocardial protection against ischaemia/reperfusion injury, stabilize electrolytes, minimize haemodilution, limit the incidence of arrhythmia, and be cost effective. In the absence of adequately powered randomized trials, there is ongoing debate over the ideal cardioplegia solution. In the last decades, single-shot cardioplegia solutions including HTK, also referred to as Custodiol®, as well as DN are widely used in contemporary adult cardiac surgery (6–11). HTK is a hyperpolarizing, intracellular, crystalloid cardioplegia solution that provides safe cardioplegic arrest for up to 2 h after a single administration. Next to its three eponymous ingredients histidine (pH buffer), tryptophan (protection of cell membranes), and ketoglurat (membrane stabilization and substrate for anaerobic metabolism), its key characteristic is a low sodium concentration inducing hyperpolarization of the myocyte plasma membrane thereby inducing cardiac arrest in diastole. DN is a depolarizing, extracellular, crystalloid cardioplegia solution and its main characteristic is a high potassium concentration leading to membrane depolarization and subsequent cardiac arrest. The hallmark component of DN is lidocaine, which is an inhibitor of the sodium potassium pump. Lidocain inhibits intracellular calcium accumulation, stabilizes cardiomyocyte cell membranes, preserves intracellular high-energy phosphates, scavenges free radicals, and buffers acid-base imbalances (15).

The majority of retrospective observational studies reported either beneficial effects of DN compared to HTK or equal cardioprotective effects (6–10). A randomized controlled trial including 125 patients undergoing minimally invasive aortic valve replacement with an ischaemic time <90 min observed no differences regarding routine biochemical measurements and basic perioperative clinical outcomes (11). The reasons for these discrepancies between a large body of evidence derived from real-world data and a relatively small randomized trial remain unclear. To the best of our knowledge, the current study is the first large animal model performing a detailed comparison of DN to HTK aiming to give detailed insight into potential histological, functional, and serological mechanisms. Due to the highly invasive nature including multiple biopsies (myocardial and vascular) as well as repeat invasive assessment of LV function not feasible in humans, translational research using large animal models are of particular clinical importance in cardiac surgery in general and specifically in studies elucidating the differing effects of cardioplegic solutions (16).

The current study observed a better functional capacity with respect to both systolic as well as diastolic properties as assessed by pressure-volume loops following DN. There were no differences regarding parameters indicative of direct ischaemia/reperfusion injury including histomorphological findings, troponin I and CK-MB, extent of oxidative and nitrosative stress, mitochondrial function, or apoptosis-inducing factors. However, calcium overload has a strong impact on myocardial recovery following cardioplegia. Therefore, one might speculate that the improved functional capacity is a consequence of depolarizing cardiac arrest with a simultaneous block of sodium and calcium influx as well as stabilization of cardiomyocyte cell membranes, which are influenced by the key components of DN including high potassium concentration and lidocaine.

A main finding of the current study is a higher rate of spontaneous return to sinus rhythm in DN. We could observe equal levels of potassium in DN vs. HTK, despite the fact that DN is a depolarizing cardioplegia using high levels of potassium. Further, sodium, calcium, and chloride concentrations remained stable in DN, whereas they decreased in HTK. Although electrolyte levels remained within the reference range in both groups, electrolyte imbalances in the reperfusion period appear to have pronounced effects on a cellular level (17). In addition, calcium influx also triggers reperfusion fibrillation. Therefore, higher electrolytical stability and combined with the inhibition of the calcium influx mainly induced by lidocaine could explain the higher rate of spontaneous return to sinus rhythm in DN. This observation was in accordance with previous real-world studies (7, 8).

In comparison to blood cardioplegia, crystalloid cardioplegic solutions reduce haemoglobin and increase the transfusion rate. In the current study, haemoglobin decrease was more pronounced in the HTK group. This can be attributed reduced haemodilution due to the composition of DN vs. HTK (cardioplegia:blood ratio 4:1 vs. pure cardioplegic solution) and total cardioplegic volume. It remains unclear if the differences regarding haemodilution are clinically relevant or how subsequent ultrafiltration and/or need for transfusions would impact clinical outcome.

Preservation of endothelial function and/or minimizing endothelial dysfunction is an important aspect of myocardial protection. Electrophysiologic *in vitro* observations suggested that hyperpolarizing cardioplegia is superior to depolarizing cardioplegia in terms of endothelial function in the coronary microcirculation (18). Similarly, Xue et al. reported a better endothelial protection by HTK when compared to DN in a rat model (19). The current study does not support the hypothesis of superiority of HTK with regarding endothelial function. We observed a lower percentage of endothelial cells with ICAM-1 expression in the DN group, which contributes to inflammation, leukocyte adhesion, and vascular permeability ultimately triggering endothelial dysfunction (20).

In summary, we observed beneficial effects of DN in comparison to HTK. Their individual clinical relevance remains unclear. However, a successful procedure and subsequently improved outcomes is often a result of multiple small positive effects. While financial aspects should not be of greater importance than efficiency and/or safety, the mean costs of <10 € per DN dose are a strong argument in favour of DN. Further research is warranted to identify the ideal cardioplegia concept.

This animal study has some limitations. First, we included healthy pigs with a healthy myocardium and no relevant comorbidities. In contrast, adult humans who require cardiac surgery most likely display pathological myocardial and vascular alterations. Although findings in pig models cannot be extrapolated to adult humans, the anatomic and cardiovascular similarities between human and pig allow a thoughtful translation of the results. Second, the concept of the current pig model did not allow further analyses of long-term outcomes. Despite this inherent disadvantage of the experimental setting, there are potential advantages such as detailed mechanistic insights as well as exclusion of external factors and environmental conditions increasing the biological variability of the outcome. Third, the DN cardioplegic solution used in this study based on Jonosteril Free Flex solution, because the originally used Plasmalyte solution has no market authorization in Germany. Therefore, the clinically used modified DN cardioplegic solution bases on Jonosteril Free Flex, too. According to the widely comparability of the ingredients, there is no reason the assume differences between both DN solutions. Fourth, at baseline, troponin I and CK-MB were increased and showed a large heterogeneity between the animals in each group. The reasons could not be detected. Fifth, we assessed hypoxic effects and mitochondrial damage using surrogate parameters such as HIF-1*α* or the green fluorescence/red fluorescence ratio after JC-1 staining. Although these are indirect markers, they have been demonstrated to correlate with mitochondrial damage (21, 22). Sixth, the surgical team could not be blinded with respect to cardioplegic solution used. However, all other investigators involved in data and sample analyses were blinded to treatment allocation. Finally, the results are limited to 90 min cardioplegic arrest and cannot be extrapolated to longer ischaemic duration. Additional studies are warranted in longer procedures.

In conclusion, in this porcine model with prolonged ischaemia, DN was superior to HTK with respect to blood electrolytes, haemoglobin levels, spontaneous return of sinus rhythm, LV function, and endothelial injury. Histomorphological parameters indicative of ischaemia/reperfusion injury, oxidative stress and mitochondrial function as well as apoptosis-inducing factors did not differ between DN and HTK. There was no indication for any inferiority of DN as compared to HTK.

## Data Availability

The original contributions presented in the study are included in the article/Supplementary Material, further inquiries can be directed to the corresponding author.

## Appendix

**Table A1:** Pressure-volume measurements at baseline.

	HTK (*n* = 9)	DN (*n* = 9)	*p* value	HTK (*n* = 9)	DN (*n* = 9)	*p* value
	4 µg/kg INN			8 µg/kg INN		
Systolic measurements						
V_Pes100_	44 ± 33 ml	67 ± 34 ml	0.19	24 ± 19 ml	46 ± 23 ml	0.06
PRSW	78 ± 28 mmHg	64 ± 17 mmHg	0.06	105 ± 39 mmHg	83 ± 21 mmHg	0.44
Diastolic measurements						
V_Ped10_	142 ± 57 ml	239 ± 118 ml	0.06	191 ± 157 ml	249 ± 136 ml	0.01
Tau	22.8 ± 2.6 ms	25.0 ± 1.7 ms	0.26	20.2 ± 2.2 ms	23.1 ± 1.7 ms	0.21

Data presented baseline measurements before the initiation of cardiopulmonary bypass under INN doses of 4 µg/kg and 8 µg/kg. Values are mean ± standard deviation. DN, Del Nido cardioplegia; HTK, bretschneider histidine-tryptophan-ketogluterate cardioplegia; INN, norepinephrine; PRSW, preload recruitable stroke work; V_Pes100_, index of end-systolic pressure-volume relationship extrapolated volume at 100 mmHg; V_Ped10_, index of the end-diastolic pressure-volume relationship extrapolated volume at 10 mmHg; Tau, rate of pressure decay during isovolumetric relaxation.
