# Supplementary material for: Comparison of Del Nido and histidine-tryptophan-ketoglutarate cardioplegia solutions: an animal study with prolonged ischaemia
Source: Front Cardiovasc Med. 2024 Dec 17;11:1457770. doi: 10.3389/fcvm.2024.1457770 (PMC11686485; doi:10.3389/fcvm.2024.1457770)
Supplement: Supplementary file 1 [file Supplementaryfile1.pdf]

Supplementary Material

Comparison of Del Nido and histidine-tryptophan-ketoglutarate cardioplegia solutions: an animal study with prolonged ischaemia

Alexandro Hoyer, Maja-Theresa Dieterlen*, Jagdip Kang, Hanna Oetzel, Karoline Wiesner, Kristin Klaeske, Philipp Kiefer, Susann Oßmann, André Ginther, Martin Kostelka, Suzanne de Waha, Michael A. Borger

*** Correspondence:** PD Dr. rer. nat. Maja-Theresa Dieterlen; mdieterlen@web.de

1 Supplementary Data

Procedural details of the experimental pig model with prolonged ischaemia

Anaesthesia. Before transportation, the pigs were sedated using intramuscular midazolam 0.5 mg/kg, atropine 0.02 mg/kg and ketamine 15 mg/kg. After establishing venous access via the lateral ear vein, propofol was administered intermittently until intubation and mechanical ventilation (Cato, Draeger, Lübeck, Germany) was established. Afterwards, continuous sedation using 25-35 mg/kg/h propofol and 0.5-2 mg/kg/h sufentanil was established. The pigs were ventilated with 33-100 Vol.% oxygen, a respiratory rate between 15-30 per min and a tidal volume of 6-10 mL/kg bodyweight. Arterial blood pressure was invasively measured via a 6F PiCCO® Catheter (PULSION Medical Systems, Feldkirchen, Germany) in the femoral artery. An 8F venous sheeth was placed in the internal jugular vein for the placement of a Swan-Ganz catheter for haemodynamic measurements and a central line was placed for the administration of medication. A 6F arterial sheeth was placed in the common carotid artery for introduction of a conductance catheter for pressure-volume loop (PVL) measurements. Blood pressure, pulmonary artery pressure, electrocardiography, body temperature, oxygen saturation, and respiratory parameters were recorded. Blood gases, pH and electrolyte concentrations were measured in blood samples using an ABL90 flex (Radiometer, Willich, Germany). Peripheral vascular resistance and stroke volume were recorded by PiCCO plus (PULSION Medical Systems).

Surgical technique, extracorporeal circulation and perfusion. Following midline sternotomy and pericardiotomy, the inferior vena cava was dissected and a vessel loop was placed around it. Heparin 300 IU/kg was administered intravenously and cannulation sutures were placed in the aortic arch and right atrial appendage. For the establishment of cardiopulmonary bypass (S III, Stöckert, Sorin Group; Milan, Italy and EUROSETS, Medolla, Italy) arterial (25 cm x 22 F, FemFlex™, Edwards Lifesciences, Irvine, USA) and venous (29 cm x 29F Trim-Flex Dual Stage Venous Drainage Cannula, Edwards Lifesciences, Irvine, CA, USA) cannulae were inserted. A needle vent (12GA DLP®, Medtronic, Minneapolis, MN, USA) for infusion of cardioplegia was placed in the ascending aorta. The conductance catheter was advanced through the common carotid artery into the left ventricle using fluoroscopy. Before cross-clamping of the aorta and infusion of the cardioplegic solution, all animals were equilibrated for 40 min. During this time baseline variables for hemodynamic measurements, PVL measurements and myocardial temperature in the interventricular septum, the left ventricular free wall and the dorsal left ventricle were documented.

Animals were then randomly assigned (n = 9 per group) to receive either HTK (Dr. Franz Köhler Chemie, Bensheim, Germany) or DN cardioplegia. The DN cardioplegic solution consisted of a 1:4

mixture of blood and Jonosteril Free Flex solution (1000 mL), mannitol 15% (21.3 mL), magnesium sulfate 50% (4.0 mL), sodium bicarbonate 8.4% (13.0 mL), potassium chloride 14.9% (13.0 mL), xylocitin 2% (3.9 mL). The application volume was set to 1800 mL HTK solution and 1300 mL DN solution. Following the application of the cardioplegic solution, the hearts were arrested for 90 min at 34 °C body temperature. During CPB, haemofiltration was performed to minimise the risk of volume overload. The aortic cross-clamp was removed after 90 min of ischaemia and following 30 min of reperfusion, the animals were weaned from CPB within 15 min. Following 120 min of reperfusion, the final PVL measurements were performed and serological samples as well as myocardial biopsies from the left and right ventricle acquired.

2 Supplementary Figures

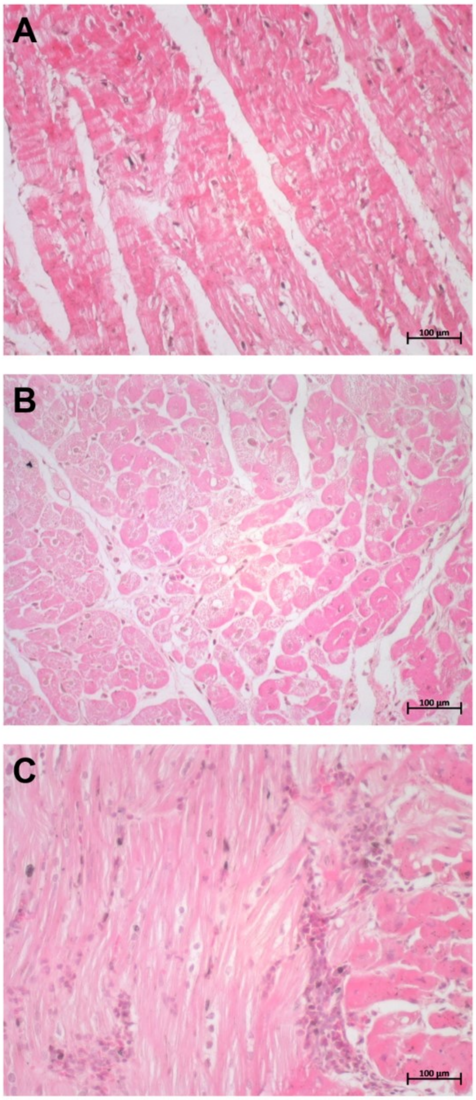

Supplementary Figure 1: Exemplary haematoxilin/eosin-stained myocardial sections. Myocardial damage can be detected by the loss of cross striation or cell borders and less colourability of nuclei (A). Edematous changes include extracellular matrix disruption of the myocardial tissue (B). Infiltrating cells can be detected throughout the myocardium (C). Scale bar = 100 µm.

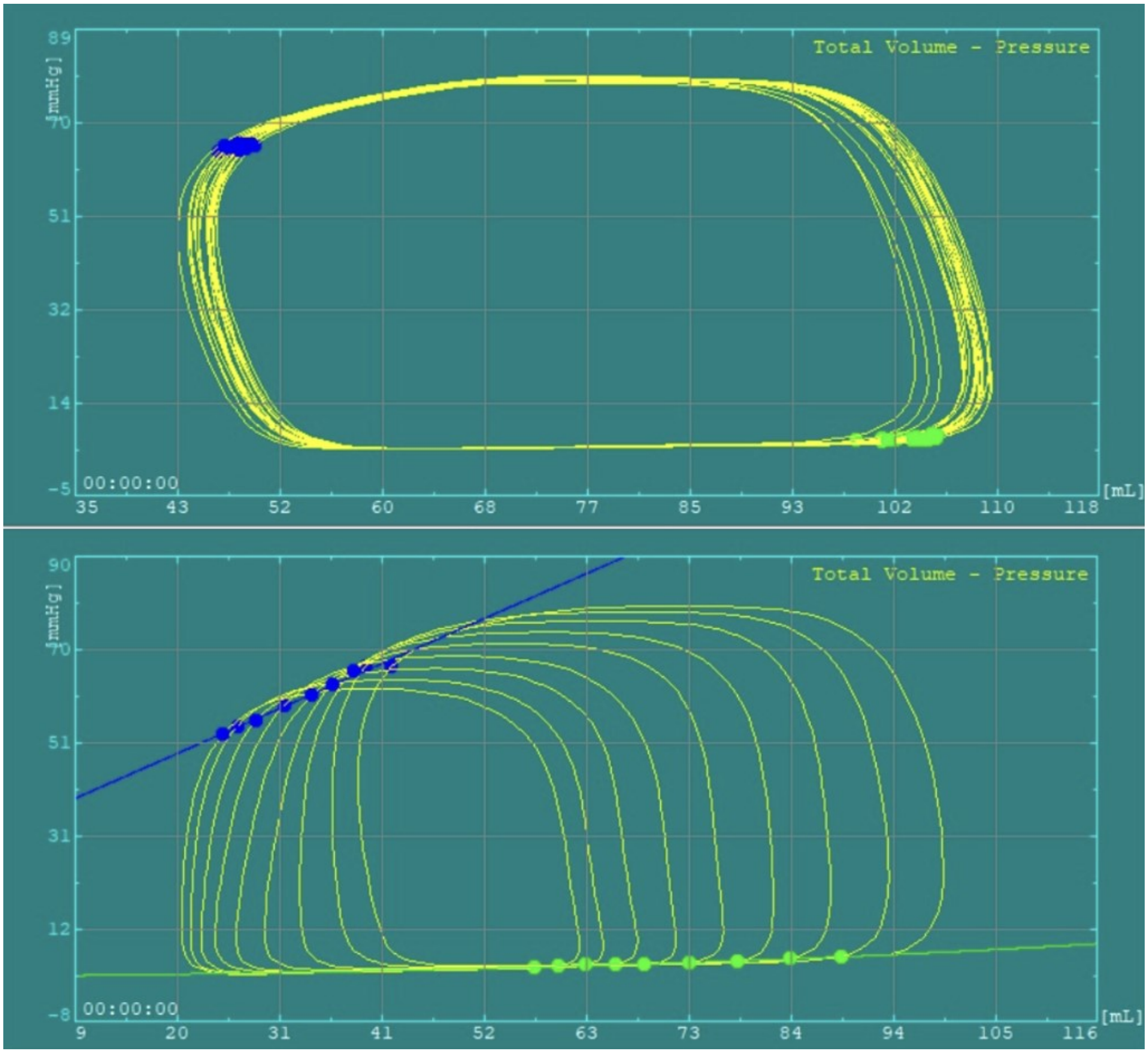

Supplementary Figure 2: Recorded pressure volume loops during breathhold (upper) and under breathhold and occlusion of the inferior vena cava.

Supplementary Table 1: Pressure-volume measurements at baseline.

	HTK (n = 9)	DN (n = 9)	p value	HTK (n = 9)	DN (n = 9)	p value
	**4 µg/kg INN**			**8 µg/kg INN**		
Systolic measurements						
V_{Pes100}	44 ± 33 mL	67 ± 34 mL	0.19	24 ± 19 mL	46 ± 23 mL	0.06
PRSW	78 ± 28 mmHg	64 ± 17 mmHg	0.06	105 ± 39 mmHg	83 ± 21 mmHg	0.44
Diastolic measurements						
V_{Ped10}	142 ± 57 mL	239 ± 118 mL	0.06	191 ± 157 mL	249 ± 136 mL	0.01
Tau	22.8 ± 2.6 ms	25.0 ± 1.7 ms	0.26	20.2 ± 2.2 ms	23.1 ± 1.7 ms	0.21

Footnote Supplementary Table 1: Data presented baseline measurements before the initiation of cardiopulmonary bypass under INN doses of 4 µg/kg and 8 µg/kg. Values are mean ± standard deviation. DN, Del Nido cardioplegia; HTK, Bretschneider histidine-tryptophan-ketogluterate cardioplegia; INN, norepinephrine; PRSW, preload recruitable stroke work; V_{Pes100}, index of end-systolic pressure-volume relationship extrapolated volume at 100 mmHg; V_{Ped10}, index of the end-diastolic pressure-volume relationship extrapolated volume at 10 mmHg; Tau, rate of pressure decay during isovolumetric relaxation.
